# Group A Streptococcus, Acute Rheumatic Fever and Rheumatic Heart Disease: Epidemiology and Clinical Considerations

**DOI:** 10.1007/s11936-017-0513-y

**Published:** 2017-03-11

**Authors:** Liesl J. Zühlke, Andrea Beaton, Mark E. Engel, Christopher T. Hugo-Hamman, Ganesan Karthikeyan, Judith M. Katzenellenbogen, Ntobeko Ntusi, Anna P. Ralph, Anita Saxena, Pierre R. Smeesters, David Watkins, Peter Zilla, Jonathan Carapetis

**Affiliations:** 1 Department of Paediatric Cardiology, Faculty of Health Sciences, Red Cross War Memorial Childrens Hospital, University of Cape Town, Cape Town, South Africa; 2 Institute of Child Health, Red Cross War Memorial Childrens Hospital, Room 2.17 2nd floor, Klipfontein Road, Mowbray, Cape Town, 7700 South Africa; 3 Department of Medicine, Faculty of Health Sciences, Groote Schuur Hospital, University of Cape Town, Cape Town, South Africa; 4 Children’s National Health System, 111 Michigan Avenue NW, Washington, DC 20010 USA; 5 Ministry of Health and Social Services, Windhoek, Namibia; 6 Department of Cardiology, All India Institute of Medical Sciences, New Delhi, India; 7 Telethon Kids Institute, Roberts Road, Subiaco, Perth Australia; 8 University of Western Australia, Crawley, Australia; 9 School of Population Health, University of Western Australia, Crawley, Perth, WA Australia; 10 Division of Cardiology, Department of Medicine, University of Cape Town and Groote Schuur Hospital, Cape Town, South Africa; 11 Global and Tropical Health, Menzies School of Health Research, Darwin, NT Australia; 12 Department of Medicine, Royal Darwin Hospital, Darwin, NT Australia; 13 Paediatric Department, Academic Children Hospital Queen Fabiola, Université Libre de Bruxelles, Brussels, Belgium; 14 Molecular Bacteriology Laboratory, Université Libre de Bruxelles, Brussels, Belgium; 15 Department of Paediatrics, The University of Melbourne, Parkville, VIC Australia; 16 Group A Streptococcus research group, Murdoch Children’s Research Institute, Parkville, VIC Australia; 17 Division of General Internal Medicine, University of Washington, Seattle, WA USA; 18 Christiaan Barnard Division of Cardiothoracic Surgery, Faculty of Health Sciences, University of Cape Town, 7925 Obsrvatory, Cape Town, South Africa; 19 Cardiovasular Research Unit, University of Cape Town, Anzio Road, 7925 Cape Town, South Africa; 20 Princess Margaret Hospital for Children, Perth, WA Australia

**Keywords:** Group A streptococcus, Acute rheumatic fever, Pathogenesis, Global burden of disease, Echocardiography, Benzathine penicillin

## Abstract

Early recognition of group A streptococcal pharyngitis and appropriate management with benzathine penicillin using local clinical prediction rules together with validated rapi-strep testing when available should be incorporated in primary health care. A directed approach to the differential diagnosis of acute rheumatic fever now includes the concept of low-risk versus medium-to-high risk populations. Initiation of secondary prophylaxis and the establishment of early medium to long-term care plans is a key aspect of the management of ARF. It is a requirement to identify high-risk individuals with RHD such as those with heart failure, pregnant women, and those with severe disease and multiple valve involvement. As penicillin is the mainstay of primary and secondary prevention, further research into penicillin supply chains, alternate preparations and modes of delivery is required.

## Introduction

Acute rheumatic fever (ARF) and its sequel, rheumatic heart disease (RHD), cause significant morbidity and mortality in developing countries, yet they are under-recognized as global health problems [1]. A recent surge in the scientific exploration of ARF and RHD has resulted in alternate hypotheses regarding the pathogenesis of ARF, new global burden of disease estimates and revised diagnostic criteria. These scientific advances have been mirrored by the declaration of their commitment to end ARF/RHD on the part of international agencies, such as the World Heart Federation (WHF) [2], the World Health Organization (WHO) [3], and the African Union (AU) [4]. This review summarizes these findings and provides a clinical perspective on ARF/RHD pathogenesis, epidemiology, diagnosis, prevention, management and control.

## Pathophysiology

Although epidemiological and immunological studies have clearly identified group A β-hemolytic streptococcus (GAS) as the etiologic agent triggering ARF in a susceptible host, the molecular pathways linking GAS to ARF are still poorly understood. Molecular mimicry and autoimmunity probably play a pivotal role in the pathogenesis of ARF and carditis [5] since it was shown that the streptococcal M protein shares an α-helical coiled structure with cardiac proteins such as myosin and that antibodies isolated from ARF patients cross-react with both M protein and heart tissue. Elevated in patients with valvular involvement, these antibodies are significantly reduced after surgical removal of inflamed valves and they correlate with poor prognosis [6]. Moreover, heart-M protein cross-reactive T cells have been isolated from the myocardium and the valves of RHD patients suggesting their involvement in the pathophysiology of the disease [7, 8]. However, the role of collagen should not be underestimated, as shown by recent studies demonstrating pathological findings in subendothelial and perivascular connective tissue in ARF [9].

It has been demonstrated that a streptococcal M protein domain called PARF (peptide associated with rheumatic fever) binds to the CB3 region of collagen type IV resulting in an antibody response to the collagen with consequent inflammation [10]. However, a recent study in New Zealand (NZ) has shown that among 74 GAS strains associated with ARF, only one GAS isolate contained the PARF motif, thus suggesting that additional and/or complementary mechanisms are likely to be involved with ARF pathogenesis [11].

At the clinical level, chronic RHD characterized by fibrinous pericarditis and interstitial granulomas or Aschoff’s nodules (loose granulomas with central fibrinoid necrosis and giant multinucleated cells) in the myocardium can resolve without residual damage while those associated with valvulitis usually lead to permanent damage [12]. This variation may be related to the healing capacity of the valvular endothelial cells [13, 14], together with exposure to collagen, either released from damaged valves or bound to GAS [15]. Once the valves are damaged, anti-collagen antibodies may well be part of the complex autoimmune response responsible for ongoing damage. Overall, these studies suggest that ARF/RHD result from a complex interplay among multiple streptococcal antigens, cross-reactive antibodies and multi-pronged immune targets.

## Current epidemiology of GAS, ARF, and RHD around the world

### Group A streptococcus

GAS causes a range of human infections, the most common of which is pharyngitis in children 5–15 years of age. GAS impetigo remains a common childhood infection in tropical developing countries [16, 17]. Controversy still remains regarding the role of GAS in infections other than pharyngitis [18–20]. The epidemiology of GAS upper respiratory infection in Fiji shows an incidence of 14.7 cases per 100 child-years (95% CI, 11.2–18.8) and among African countries, proportions of sore throats due to GAS ranging from 9.3% in Morocco to 41.3% in Tunisia [21, 22].

Subtyping based on the highly variable N-terminus of the GAS surface M protein allows for characterization of GAS into >200 so-called emm-types [23, 24]. Recently, Sanderson-Smith proposed a classification scheme based on 48 emm-clusters, facilitating surveillance and the vaccines development [25]. Significant global variation in emm-type distribution has been reported, highlighting the failure to include predominant emm-subtypes from the south Pacific [26] and Africa [27] in earlier M-protein based vaccine initiatives.

### Acute rheumatic fever and rheumatic heart disease

**Fig. 1:**
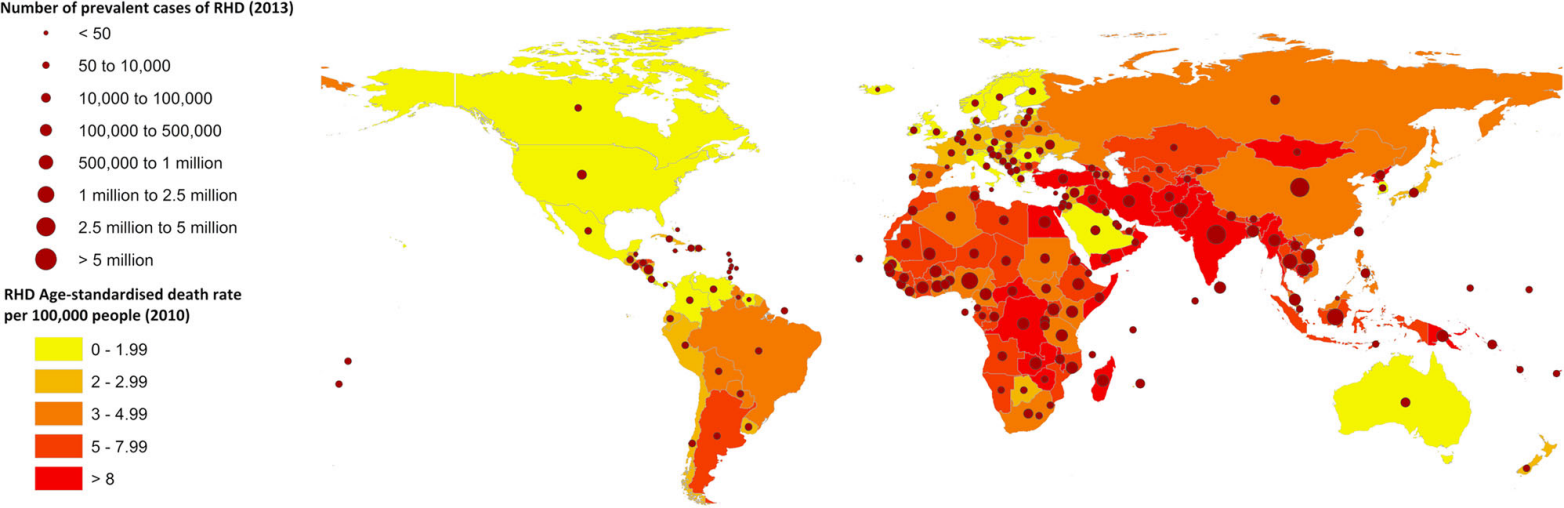
Global prevalence and mortality rates. Source: data derived from Global Burden of Disease data 2010/2013.

The incidence of ARF peaks between 5 and 15 years of age and is rare over 30 years of age, with approximately 60% of people with ARF in endemic communities subsequently developing RHD [28, 29]. ARF incidence is similar in males and females but the risk of RHD is 1.6–2.0 times greater in women [29, 30] likely due to several factors including worsening of existing disease during pregnancy [31, 32], GAS exposure during child rearing, limited access to services and intrinsic/hormonal factors [33]. The most recent estimates of the global burden of RHD include 9 million disability-adjusted life years lost, 33 million prevalent cases and 275,000 deaths each year, with deaths occurring predominantly in low- and middle-income countries (LMICs) as depicted in Fig. 1 [34–36]. RHD prevalence increases with age [29] with survival varying with access and adherence to secondary prophylaxis to prevent ARF recurrence, severity of valvular damage and access to specialist management and surgery [17, 37].

The epidemiology of RHD varies by region [17], with a particularly high prevalence in Africa [32, 38, 39] and the Pacific region [40–43] but a high burden also in Latin America [44], the Middle East [45, 46] and Asia [17, 47]. The age-distribution of prevalent RHD cases globally reflect two distinct epidemics. The first epidemic occurred until the mid-20th century in high-income countries where the majority of surviving prevalent cases is over 50 years of age with few incident cases [17, 34]. The second on-going epidemic is characterized by a very high incidence in LMICs and among disadvantaged communities living in industrialized countries [17, 34] such as minority indigenous peoples living in Australasia [29, 48–50] and North America [51–54]. This on-going epidemic is reflected in high proportions of cases at young ages, reducing with age due to poor survival. Australia is a good case study to contrast two populations experiencing different epidemics in the same country, using RHD/ARF hospital admission counts as a proxy for burden in Australia. RHD is also associated with almost a quarter of prevalent Indigenous Australian stroke cases aged 20–34 years [55].

### Diagnosis and clinical features of acute rheumatic fever

ARF is a systemic inflammatory autoimmune reaction that appears 2–4 weeks after GAS pharyngitis with major manifestations including carditis (50–78%) [56–58], arthritis (35–88%), [56, 58], erythema marginatum (<6%) [59], and subcutaneous nodules (<1–13%) [58, 60]. Additionally, 2–19% [56, 61] of patients present with Sydenhams’ chorea, a neurological condition characterized by involuntary movements and behavioral changes [60]. Minor manifestations include PR prolongation, less severe joint manifestations, fever, and elevated inflammatory markers [62].

The carditis of ARF is a pancarditis, with valvulitis being the most common presentation [63]. It ranges widely in severity from mild sub-clinical involvement (16.8%) [64] to severe carditis with congestive heart failure and/or death (20%) [56]. Most cases involved the mitral valve. Isolated aortic valve disease occurs in only 2% of patients and right-sided valvulitis is seen only in combination with left [65].

The arthritis of ARF is most classically a painful large joint migratory polyarthritis. The arthritis improves dramatically with anti-inflammatory therapy and a history of self-medication prior to presentation can mask recognition. Evidence from Australia [61, 66], India [67], and the Pacific [68] has highlighted the importance of monoarthritis and polyarthralgia in the presentation of ARF, and the most recent diagnostic criteria now take these presentations into account [62].

Sydenham’s chorea occurs 1–8 months after GAS infection [69]. Choreiform movements are non-rhythmic, involuntary, often asymmetrical, and disappear with sleep. Concurrent muscular weakness and emotional disturbances (crying, restlessness, obsessive-compulsive symptoms, rare psychosis) can occur [70]. Alternate diagnoses should be excluded. Evidence of recent GAS infection and/or carditis provides supporting evidence of ARF, but is not needed to make the diagnosis [71].

### The 2015 Jones criteria

ARF remains a clinical diagnosis, with no single confirmatory test. The most widely used diagnostic criteria (The Jones Criteria) were first proposed in 1944 (Jones, 1944), and were recently modified in 2015 [62]. Diagnosis with an initial episode of ARF requires documentation of a recent streptococcal infection and at least two major or one major and two minor criteria (see Table 1). The 2015 Jones criteria include several important changes to previous iterations including the inclusion of echocardiography for the diagnosis of carditis, two sets of criteria based on risk stratification, and the provision of specific recommendations for diagnosis of recurrent ARF.

**Table 1:**
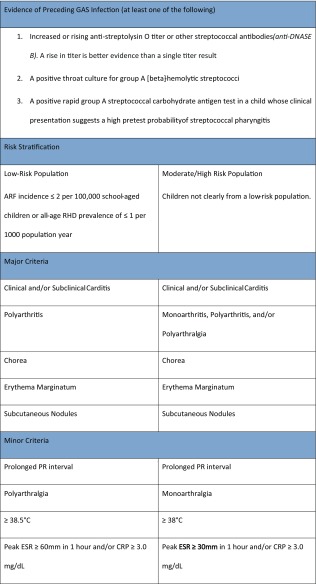
Summary of the 2015 Jones criteria [62]

Sub-clinical carditis (SCC) is now recognized as a manifestation of ARF. A meta-analysis of 23 studies from 5 continents demonstrated that 16.8% of patients with ARF had SCC and that almost half of these (44.7%) showed progressive valve disease [64]. Thus, the 2015 Jones criteria place a high importance on echocardiography and provide specific diagnostic criteria for SCC. Clinical or SCC fulfills a major criterion in all populations. It is recommended that, when possible, all patients with confirmed or suspected ARF undergo echocardiography to evaluate for carditis, with those who are negative on first evaluation undergoing repeated study to assess for evolving cardiac disease [62]. Additionally, echocardiography is considered more specific than auscultation [72], and a normal echocardiogram excludes the diagnosis of clinical carditis [62].

The 2015 Jones criteria distinguish low-risk populations, where it is important to avoid over-diagnosis of ARF, from moderate-to-high risk populations, where it is most important to ensure ARF is not missed [62]. Clinicians are directed to the moderate-to-high risk pathway if a suspected ARF patient is not clearly from a low-risk group (ARF incidence <2 per 100,000 school-aged children per year or an all-age prevalence of RHD of ≤1 per 1000 population per year).

Patients with ARF are at high risk for recurrent ARF. Patients with a history of ARF/RHD, documentation of a recent streptococcal infection, and exclusion of competing diagnoses should be diagnosed with recurrent ARF when 2 major, 1 major and 2 minor, or 3 minor criteria are fulfilled [62].

### Management of acute rheumatic fever

Management of ARF involves controlling inflammation, managing carditis, eradicating GAS and preventing recurrences (see Table 2). Aspirin has been the mainstay of anti-inflammatory treatment and is most commonly used in practice. Naproxen may have equivalent efficacy with fewer side effects [73]. In cases of severe rheumatic carditis, most clinicians believe the addition of glucocorticoids has therapeutic benefit, but contemporary studies are lacking. Other anti-inflammatory agents (e.g., ACTH, immunoglobulins) have not been shown to be superior in modification of carditis and are not routinely recommended.

Patients with severe carditis should also be managed with conventional heart failure therapy. Bed rest for those with moderate-to-severe carditis is still recommended but there is a lack of contemporary data supporting this practice [74]. Surgery during the acute phase is avoided when possible and is associated with poor outcomes [75]. Pharyngeal eradication of GAS is important and is universally recommended [76]. Oral or intramuscular penicillin is most commonly utilized. Symptomatic household contacts should also have throat cultures with treatment for positive results [76].

Sydenham’s chorea resolves in an average of 12–15 weeks, but symptoms can last for years, and recurrences are seen in up to one-third of those [77]. Treatment for Sydenham’s chorea is recommended when associated with significant motor impairment. Corticosteroids may shorten the disease course but do not change the rate of relapse. Other typical and atypical antipsychotics and neuroleptic medications are reserved for resistant patients [78]. As with other ARF presentations, eradication of GAS and initiation of secondary penicillin prophylaxis are recommended, and recurrent GAS infection is associated with recurrences [79].

### Comprehensive evaluation in rheumatic heart disease

#### Echocardiography

Echocardiography is indispensible for assessment of valve lesions secondary to RHD and the gold standard comprehensive assessment prior to surgical or catheter intervention. Transesophageal echo may be required in some cases, especially in older patients to better define features of RHD.

The most common lesion is mitral regurgitation (MR), while mitral stenosis (MS) is (outside of congenital mitral stenosis) pathognomonic of RHD.

**Fig. 2:**
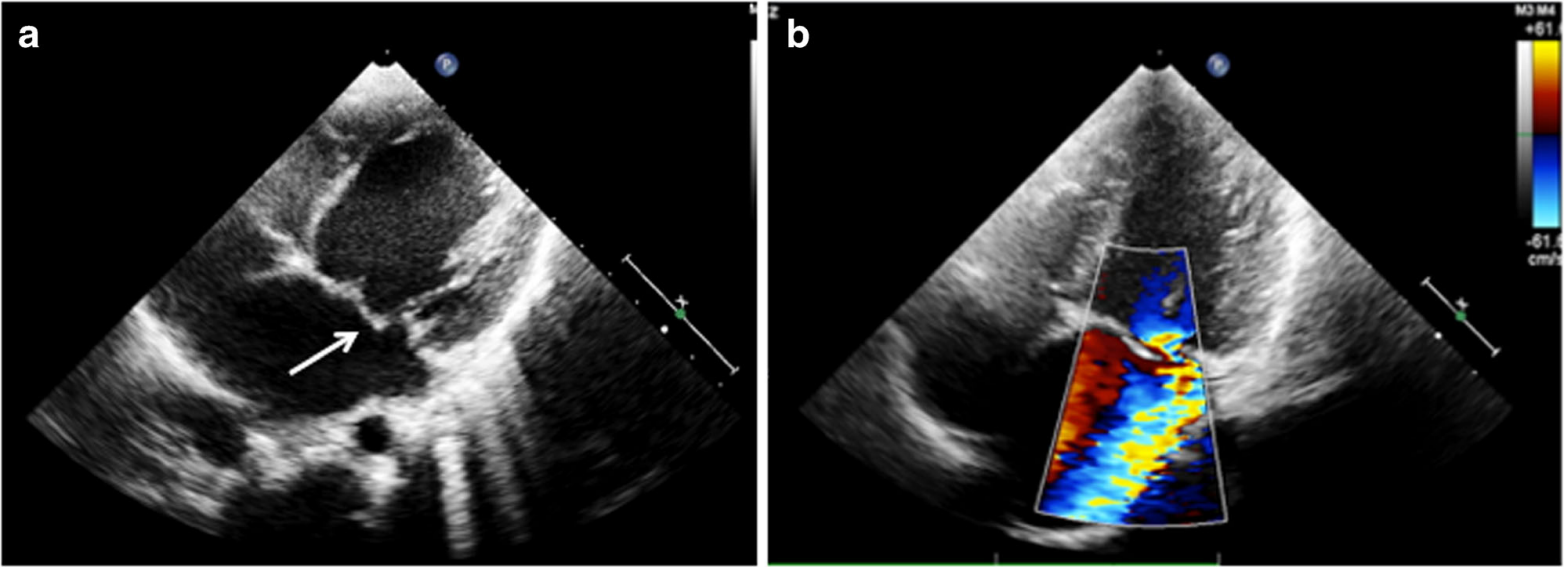
Echocardiogram from a patient with severe mitral regurgitation secondary to a flail anterior mitral leaflet (*arrow*) (**a**). Color Doppler shows severe eccentric mitral regurgitation (**b**).

In MR, two-dimensional echocardiography (2DE) shows elongated chordae causing prolapse of the anterior mitral valve leaflet (AML). In acute cases, either due to acute rheumatic carditis or superimposed infective endocarditis, chordal rupture may occur, seen as flail AML along with severe eccentric MR (Fig. 2). Color flow imaging can grade the severity of MR. Depending on MR severity and chronicity, the left atrium and left ventricle dilate. Left ventricular end systolic and end diastolic volumes, and ejection fraction can be calculated using Simpson’s or area-length methods.

**Fig. 3:**
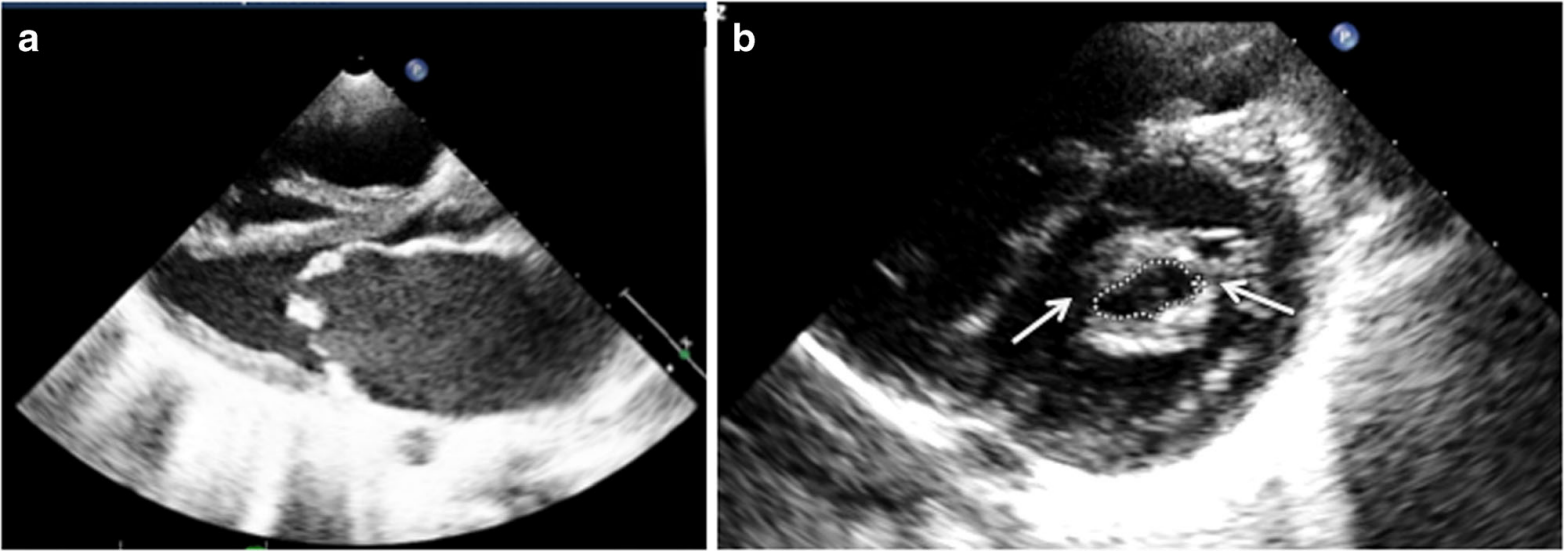
Echocardiogram from a patient with severe mitral stenosis showing thickened mitral valve with restricted opening (**a**) and commissural fusion (*arrows*) (**b**). Mitral valve area by planimetry is 0.73 cm^2^.

In patients with MS, 2DE shows commissural fusion and chordal thickening. The restricted movement of mitral leaflets results in doming, producing elbow or dog leg deformity of the anterior mitral leaflet (AML). Often the posterior mitral leaflet is also rigid and shows paradoxical motion (Fig. 3a). Calcification of valve leaflets may be seen in older patients. Color Doppler shows a narrow diastolic jet that may be eccentric due to significant subvalvular deformity. Severity of MS can be assessed by calculating mitral valve area, most commonly by Doppler derived pressure half time and 2DE-derived planimetry methods (Fig. 3b) [80]. The left atrium among MS patients is enlarged; in some cases, it may show a thrombus in the cavity or in its appendage. Tricuspid regurgitation with dilation of right sided chambers is seen in those with significant pulmonary hypertension secondary to severe MS.

Aortic valve involvement due to RHD is often associated with MR, MS or both. Although aortic regurgitation (AR) is more common than aortic stenosis, both may coexist. The valve leaflets will be thickened, especially at level of their edges. Commissural fusion with restriction of leaflet motion is quite pathognomonic of chronic rheumatic process. In patients with AR, coaptation defect and leaflet prolapse are easily demonstrable on echocardiography. Color flow imaging quantifies the severity of AR [81]. The left ventricle is enlarged in significant and chronic AR; left atrium size is usually normal unless MR is associated.

Three dimensional echocardiography (3DE) allows for the visualization of the entire mitral valve apparatus and assessment of MS and MR severity. Real time 3DE color Doppler imaging measures Proximal Isovelocity Surface Area (PISA) without geometric assumptions or the requirement of an angle correction factor [82]. Similarly, the cross-sectional area of vena contracta is more accurately determined by 3DE [83]. The mechanism of MR as shown on 3DE helps the surgeon to predict the complexity of repairing the valve [84]. The role of 3DE is expanding for aortic valve lesions; it is likely to be useful for guiding valve repair.

#### Portable and handheld echocardiography

The use of portable echocardiography was demonstrated in the first screening studies of asymptomatic schoolchildren [85, 86] with comparable color, Doppler and morphological assessments. These portable echo machines provide highly needed technology at a fraction of the price of larger machines. As the need for decentralized and inexpensive echocardiography has grown, particularly in RHD-endemic countries, hand-held or ultra-portable machines have been applied very successfully in clinical practice and research [87]. Despite the limitations of these machines, the ability to cheaply and accurately visualize a rheumatic valve, assess left ventricular function and exclude pericardial effusions in remote settings has tremendous value [88, 89].

#### Cardiac catherization

Cardiac catheterization is rarely required for making a diagnosis or informing treatment decisions. There are however a few situations in which information from catheterization and angiography may be required for planning appropriate treatment. Firstly, older patients scheduled for valve surgery require coronary angiography to rule out the presence of significant coronary artery disease (CAD). In general, men with RHD over the age of 40 years and women over the age of 50 years should undergo coronary angiography before valve surgery [90]. Secondly, RHD involves multiple valves in a large proportion of patients [91]. Assessment of lesion severity in the presence of concomitant other valve disease may be problematic using echocardiography. For example, the presence of severe AR or left ventricular diastolic dysfunction may underestimate the severity of MS as measured by Doppler pressure-half-time methods [92]. Direct measurement of the transmitral gradient at catheterization may help in better assessing the severity of MS. Left ventricular and aortic root angiography may also supplement information on regurgitation severity obtained at echocardiography in patients with multiple stenotic and regurgitant lesions. Finally, direct measurement of pressures, transvalvular gradients and blood flow may sometimes be necessary when there is discrepancy between symptoms, clinical findings and echocardiography. Cardiac catheterization data may thus help fine-tune treatment decisions.

#### Magnetic resonance imaging

Multiparametric cardiovascular magnetic resonance (CMR) combines a variety of transmitted radiofrequency pulses and magnetic gradients in the presence of a powerful magnetic field (pulse sequences) for a comprehensive assessment of RHD. An advantage of CMR over echocardiography in RHD is the ability to provide accurate and reproducible information on tissue characteristics including myocardial fibrosis and edema [93, 94]. Unlike echocardiography, CMR imaging does not depend on the presence of adequate acoustic windows and operator experience for consistently obtaining interpretable images, and may provide additional diagnostic information when echocardiographic imaging is suboptimal [95, 96]. The limitations of CMR evaluation of valve disease include its current relative inability to be used in patients with certain types of metallic implants. All prosthetic valves are safe for CMR imaging; however, they produce local artifact; hence, the ability of CMR to assess the detailed structure of a prosthetic valve may be limited. Although CMR could provide important research information, it is a rare and expensive resource in RHD-endemic countries and data regarding prognostic significance of CMR in RHD are sparse [97].

### Interventions

#### Catheter-based interventions

Percutaneous balloon mitral valvotomy (PBMV) is the procedure of choice in patients with severe or symptomatic MS. Scores incorporating adverse morphological features of the valve and sub-valvular apparatus determine suitability for PBMV [98]. Among suitable patients without atrial fibrillation or other contraindications, randomized trials have demonstrated durable acute and intermediate-term outcomes that are not different from surgical commissurotomy [99, 100]. There is also limited experience with balloon valvotomy in aortic and tricuspid valve stenosis [101]. In general, however catheter-based interventions can only be used in predomnantly stenotic lesions.

#### Cardiac surgery

Once symptomatic, cardiac surgery becomes the only life-saving option for the majority of patients with RHD, yet this is largely not available in RHD-endemic areas. In Africa, this lack of access is most glaring. The 900 million living in Sub-Saharan Africa outside South Africa have access to only 22 cardiac centers [102]. Even with the most conservative estimate, Africa would need another 400 cardiac centers at a capacity comparable to the existing ones.

Given the slow inroads prevention made in the past 15 years [103], surgery will remain an integral part of the treatment of RHD for a long time. Furthermore, identifying patients for surgery when they are still operable would require a significant diagnostic empowerment within resource and expertise constrained systems.

Symptomatic MR is the leading pathology presenting in the first two decades of life [104]. Up to one third of these patients already need life saving surgery for decompensated pure MR at presentation [104]. Those not requiring surgery during the acute stage often require surgery soon after. These patients have typically thin, pliable leaflets with no scarring but a significant increase in mitral annular diameter [105]. Echocardiographically, this is evident as a posteriorly directed regurgitation jet, often associated with AML prolapse [105]. In three out of four cases, the chordae tendineae are elongated and in a few even ruptured [106]. At the other end of the spectrum of aggressive disease progression stands juvenile symptomatic MS. In an Ethiopian study, one third of children under the age of 15 with symptomatic RHD had developed MS [107]. While pure MR in children has excellent results with repair [108] and pure MS shows better long-term results after balloon valvuloplasty [109] than after surgery [110], the aggressive disease progression of RHD presenting in childhood is characterized by multi-valvular involvement in a majority of patients [91, 107] requiring prosthetic surgery.

While surgical patients who participated in studies during the 1960s and 1970s were largely in their late twenties and early thirties [111], today’s patients come to surgery in their forties to early fifties in the urban populations of threshold countries (mostly upper middle-income countries) [112]. With two thirds of mitral patients over the age of forty showing degrees of leaflet thickening and rigidity [113], the repairable group is limited to a minority of the patients and even with sophisticated surgical techniques have high re-operation rates [114]. As such, prosthetic solutions need to resolve the dilemma of early degeneration in bioprosthetic valves [115] versus high thrombo-embolic complications [116] in mechanical valves for whom adherence is more challenging [117]. In view of the limited access to open heart surgery in countries where RHD is prevalent [118], simplified trans-catheter implantations of long-lasting, affordable synthetic valves may hold a key to an accessible surgical therapy of RHD [119] for those patients most affected by the disease.

### Global research consortia

#### Screening for asymptomatic RHD

If patients with RHD are diagnosed early with timely institution of secondary prophylaxis, the progression to permanent valve damage due to recurrent episodes of ARF may not occur. This argument has led to a multitude of echocardiographic screening programs to document the prevalence of subclinical disease and to institute early therapy in individuals with screen-positive lesions [120–126]. Although this has resulted in much debate [127, 128], it has also led to the publication of the WHF evidenced-based guidelines for the echocardiographic diagnosis of RHD in screening of asymptomatic populations [129]. This represents an important new contribution to the research landscape of RHD, providing a much-needed objective method to ensure uniformity in diagnosis and following participants with subclinical lesions detected in screening studies to determine the progression of these lesions [130–134]. This has also marked a new era of multinational research consortia in the RHD landscape.

#### Clinical burden of disease

The clinical burden of RHD in LMICs was highlighted in a multi-center study involving 14 countries from Africa, India and Yemen [135]. The Global Rheumatic Heart Disease Registry (REMEDY) revealed that patients were young, largely unemployed and mostly women in childbearing age with severe manifestations and complications of the disease, and that delivery of secondary prevention and tertiary interventions such as anticoagulation is poor. In a rare example of “research to action,” this data informed the development of RHD action plans in Africa [136]. This study has resulted in two further international collaborations: the RHDGen consortium, focusing on the genetic epidemiology of RHD, with similar work in Australia, NZ, Fiji and the UK, and the Investigation of Rheumatic AF Treatment Using Vitamin K Antagonists, rivoroxaban or aspirin studies (INVICTUS)—an ambitious project enrolling over 25,000 cases worldwide [137, 138].

#### RHD centers of excellence

In 2015, a national consortium of Australian RF/RHD researchers established the END RHD Center for Research Excellence (CRE). This has a singular vision to develop a costed, stepwise strategy that would lead to elimination of RHD as an issue of public health importance in Australia. This will include policy makers, service providers, and community members to undertake research and translation activities in all areas of RHD prevention and control and develop a significant program of research specifically focused on engaging people living with RHD. Similar centers of excellence are proposed in Africa and other endemic areas [139].

#### RHD Action

RHD Action was launched in 2015 as a global movement committed to reducing the burden of RHD in vulnerable populations throughout the world. It combines the scientific and technical expertise of Rheumatic Heart Disease, Evidence, Advocacy, Communication and Hope (RhEACH), the policy efforts of the WHF and the platform provided by Medtronic Foundation to provide tools [140] to strengthen efforts in-country and empower and support people living with and affected by RHD and addresses the need for multidisciplinary and intersectional collaboration in addressing RHD (http://rhdaction.org/).

## Global strategies to eliminate RHD in the twenty-first century: prevention and control programs

### Prevention

ARF risk factors at the *primordial* level are relatively well defined, with household overcrowding the most consistently identified risk [141–143]. Evidence suggests that environmental conditions are the major determinant of ARF and RHD. However, few evidence-based, cost-effective primordial preventive strategies have been identified. Strong *primary* preventive programs (active case detection and treatment of streptococcal sore throat) are in place in NZ with school-based sore throat clinics targeting populations in whom ARF rates are high [144, 145]. *Secondary* prevention is the key focus of most control strategies, due to its proven efficacy, cost-effectiveness, and the challenges of primordial and primary prevention. Secondary prevention comprises 4-weekly intramuscular benzathine penicillin injections (Table 2). This treatment regimen [146] significantly reduces ARF recurrence rates [147] and can reduce the severity of rheumatic valvular lesions, or be associated with their regression of valve lesions, over time [17, 146]. There is evidence that integrating primary and secondary prevention can be very effective and affordable in low-income settings. For instance, one study estimated that Cuba’s integrated ARF/RHD program reduced the burden of RHD by over 90% and that, following an initial investment of about US$ 0.07 per child per year, substantial treatment costs were saved in the long run [148].

**Table 2:**
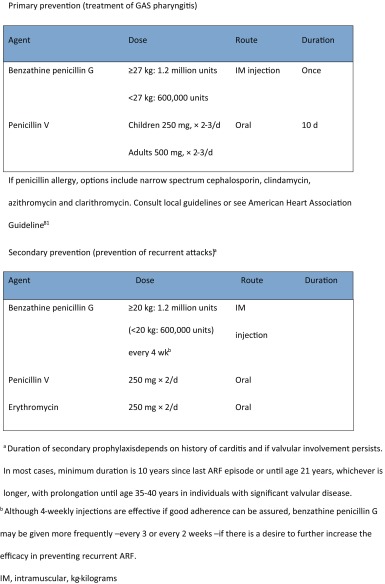
Primary and secondary prevention [163]

### Control programs

Register-based control programs are essential to guide patient care and provide a valuable epidemiological resource [149] (Table 3). Many international examples of successful register-based programs exist, including in low resource settings in Africa, Oceania and South America [150, 151]. The best measure of an effective secondary prevention program is the ARF recurrence rate. In NZ, where ARF/RHD registers have been operating for many years, documented recurrence rates are less than 10% [152]. When a register-based secondary prevention program was introduced in northern Australia in 1997 [153], around 45% of ARF cases were recurrences [154] but by 2015, this proportion had fallen to 26% (Top End RHD Control Program—unpublished data).

**Table 3:** Key roles of control programs

Key roles of control programs	
• Establish an ARF/RHD register to help coordinate care, improve delivery of long-term treatment, reduce recurrent ARF in those at risk, and follow up individuals defaulting from treatment	
• Ensure all individuals with ARF or RHD are included on ARF/RHD disease registers	
• Improve delivery of long-term secondary prevention treatment, to prevent recurrent ARF and development or progression of RHD	
• Support clinical and public health practice by increasing disease awareness and expertise among the health workforce, so that they can provide appropriate health services to people with ARF and RHD, including clinical care and follow-up, in line with best practice	
• Provide advice on education and self-management support for people with ARF and RHD and their families, and the community	

Prevention and control programs are placing increasing emphasis on the complexity of health-related issues with the resultant focus on interdisciplinary approaches. The Awareness, Surveillance, Advocacy and Prevention (A.S.A.P) program is one such initiative [155, 156], drawing on different disciplines to plan a multi-pronged tactic to attack RHD on all fronts, incorporating public health principles while advocating, backed by robust data, for community-appropriate interventions. Launched in 2006, it targets efforts to raise *Awareness*, establish *Surveillance* systems, *Advocate* for increased resources for treatment, and to promote *Prevention* strategies, using a community-based, bottoms-up approach and is an example of programs initiated from, run by and organized by countries most affected by the disease.

### Economic aspects of RHD prevention and control

#### Economic impact of RHD

A recent systematic review summarized the handful of studies on the health system costs of RHD. These ranged from US$ 2 per patient screened by echocardiography in Fiji to US$ 2900 per hospital admission for ARF and US$ 10,900 per valve surgery in South Africa. Similarly, the average yearly cost per patient with RHD in Brazil was about US$ 1500. The larger societal costs of RHD are less well characterized, though indirect costs—resulting from school and work absenteeism—occur routinely [157]. Further, there is some evidence that the economic consequences of premature death are enormous, since the majority of RHD deaths occur among children and working-age adults [158].

#### Cost-effectiveness of primary and secondary prevention

In contrast to high-income countries, where throat cultures and rapid antigen tests for GAS are widely used, a clinical decision rule threshold was found to be the most cost-effective approach to pharyngitis in South Africa [159]. These results need to be replicated in low-income countries, however, since GAS epidemiology and healthcare costs are very different in those settings.

The cost-effectiveness of passive case finding was demonstrated in a multi-country, quasi-experimental study conducted by the WHO in the late 1970s [160]. Since the advent of active echocardiography-based case finding, one analysis has suggested that this approach could be more cost-effective than passive case finding [161]. Still, more data on long-term outcomes following echocardiography screening are needed before the active approach can be recommended to policymakers.

#### Integrated health policy and initiatives

In 2015, the Social Cluster of the AU Commission hosted a consultation with RHD experts to develop a roadmap to eliminate ARF and eradicate RHD in Africa. Seven key actions are recommended for Health Ministers in the Addis Ababa Communiqué on ARF and RHD; Heads of State of member countries adopted this communiqué in June 2015 [4].

Two action points are specific to RHD; prospective RHD registers at sentinel sites and consistent availability of high-quality benzathine penicillin especially as there is concern regarding the shortage of penicillin in several countries. Other action points speak strongly for integration with further role players in primary health care and maternal and child health to improve access to reproductive health services for women with RHD who are at particular risk during the peri-partum period [162]. Failures in RHD control are often consequent to fragile health systems therefore RHD control activities should strengthen the broader healthcare system. The decentralization of technical expertise and point of care technologies to the primary and district levels will improve the recognition, diagnosis, secondary prevention, and treatment of RHD. Centers of excellence for cardiac surgery will deliver surgery for RHD with obvious benefit for the treatment of other heart diseases. It was recognized that for success we must look beyond the health sector to other custodians of the social determinants of disease. Therefore, the call for multi-sectoral integrated national RHD control programs led by the Ministry of Health but in partnership with other Ministries (such as youth, gender education, housing), academia, civil society and importantly people living with and affected by RHD.

These action points are developed for Africa but are relevant to all nations with a heavy burden of RHD where the control of this preventable disease presents a challenge to public policy. However, the eradication of RHD also requires global leadership and institutional support. There is now a call for a binding WHO resolution on global RHD control measures in 2017. An old African proverb is prescient: “If you want to walk fast, walk alone, if you want to walk far, walk together”.

## Summary and conclusions

ARF and RHD are clear indicators of fragile health structures and public health inadequacies. However, there are strong displays of initiatives that are harnessing multi-sectorial synergies to combat this disease, armed with the new global burden of disease data and revised criteria to diagnose ARF and subclinical RHD. We need to use these data for targeted interventions; identification of at-risk individuals and comprehensive prevention and control programs which contribute to resilient and effective health systems.

## Acronyms

AR: Aortic regurgitation
ARF: Acute rheumatic fever
CMR: Cardiovascular magnetic resonance
GAS: Group A β-hemolytic Streptococcus
LMIC: Low- and middle-income countries
MR: Mitral regurgitation
MS: Mitral stenosis
PISA: Proximal isovelocity surface area
RHD: Rheumatic heart disease
WHF: World Heart Federation
WHO: World Health Organization
